# Long-Term Data Reveal a Population Decline of the Tropical Lizard *Anolis apletophallus*, and a Negative Affect of *El Nino* Years on Population Growth Rate

**DOI:** 10.1371/journal.pone.0115450

**Published:** 2015-02-11

**Authors:** Jessica Stapley, Milton Garcia, Robin M. Andrews

**Affiliations:** 1 Smithsonian Tropical Research Institute, Apartado Postal 0843–03092, Panamá, República de Panamá; 2 Department of Biological Sciences, Virginia Tech, Blacksburg, VA 24061, United States of America; University of Sao Paulo, BRAZIL

## Abstract

Climate change threatens biodiversity worldwide, however predicting how particular species will respond is difficult because climate varies spatially, complex factors regulate population abundance, and species vary in their susceptibility to climate change. Studies need to incorporate these factors with long-term data in order to link climate change to population abundance. We used 40 years of lizard abundance data and local climate data from Barro Colorado Island to ask how climate, total lizard abundance and cohort-specific abundance have changed over time, and how total and cohort-specific abundance relate to climate variables including those predicted to make the species vulnerable to climate change (i.e. temperatures exceeding preferred body temperature). We documented a decrease in lizard abundance over the last 40 years, and changes in the local climate. Population growth rate was related to the previous years’ southern oscillation index; increasing following cooler-wetter, *la niña* years, decreasing following warmer-drier, *el nino* years. Within-year recruitment was negatively related to rainfall and minimum temperature. This study simultaneously identified climatic factors driving long-term population fluctuations and climate variables influencing short-term annual recruitment, both of which may be contributing to the population decline and influence the population’s future persistence.

## Introduction

Global climate change may represent the greatest threat to biodiversity in human history with an estimated one quarter of all species at risk of extinction [1]. Predicting future changes in climate, and their impacts on local, or even regional biotas, however is not simple for at least three reasons; firstly, climate change varies spatially [2–4], secondly, factors affecting population abundance are complex and include intrinsic and extrinsic factors [5,6], and thirdly, taxa vary in their vulnerability to climate change [6,7].

Despite an overall global warming trend [8], climate change is complexly related to geography and difficult to predict both in terms of magnitude and direction. In an effort to account for this inherent complexity, previous studies have used global indices of climate such as the Northern Atlantic Oscillation (NAO) or the Southern Oscillation Index (SOI) as a predictor in models attempting to link species abundance and weather. These global cycles have been found to be drivers of population abundance in many animal and plant populations (e.g. [9,10,11]). However, these global indices are not always better predictors of abundance than specific climate variables or regional variables [5] and small-scale thermal heterogeneity within habitats can also strongly influence a populations’ response to climate change and may ameliorate the effects of large-scale changes in climate [4]. To better link climate change to population abundance, studies of population change over time need to include climate at both local and global scales in their analyses.

The second reason why predicting the impact of climate change is difficult is that factors regulating populations are complex and can include both intrinsic and extrinsic factors. Understanding how long-term shifts in climate and weather influence population dynamics is a major challenge in ecology [5]. Intrinsic factors, which vary between species, such as density dependent population growth rates, are likely to influence the population response to climate change and/or obscure climate effects [5]. Extrinsic factors like predation and food availability can also complicate abundance relationships with climate. Climate can influence survival and recruitment differentially, and the susceptibility of different cohorts may differ. Knowledge of how total and cohort specific abundance is related to climate variables is likely to improve our ability to predict how climate change will effect populations.

Finally, climate change effects are difficult to predict because different taxa vary in their vulnerability to changes in climate, making general predictions difficult. Tropical ectotherms are considered at great risk from increasing temperature because they have narrow thermal tolerances [7]. In particular, lowland forest species of lizards may be most vulnerable to climate change, because they have relatively low optimal body temperatures and little opportunity to avoid warming environments [12]. Declines in abundance and local extinctions have been documented in tropical lizards and climate change has been implemented as the casual factor [13]. These studies suggest that physiological constraints inherent to a species need to be included in models attempting to understand the effects of climate change.

It follows then, that to identify how climate change affects populations we need to understand changes in local climate, consider density dependence and cohort specific relationships, and species’ intrinsic vulnerability. In this study we used 40 years of lizard (*Anolis apletophallus*, formerly *A*. *limifrons*) abundance data from Barro Colorado Island (BCI) in Panama and local and global climate data to address the following questions i) how has the local climate changed, ii) how has lizard abundance, population growth rate and annual recruitment (cohort-specific abundance) changed, and iii) how does abundance, growth rate and recruitment relate to specific climate variables; including climatic factors that may make the species vulnerable to climate change (i.e. ambient temperatures exceeding field-preferred body temperature). We document changes in the climate on BCI, mostly consistent with expectations. Lizard population abundance has fluctuated over the last 40 years but we identified an overall decline in lizards on BCI. Population growth rate was positively related to southern oscillation index (*la niña* phase) in the previous year. Annual recruitment measured indirectly as cohort specific abundance was negatively related to rainfall and minimum temperature that lizards experience during that year.

## Methods

### Study species and census

**Ecology and life history.** *Anolis apletophallus* is a small arboreal lizard of the forest understory, with essentially an annual life cycle; eggs hatch in ∼44 days [14], hatchlings grow quickly and reach maturity (44mm) within 4–6 months [15] and adult survival is less than 5% [16]. Thus eggs of one year are the adults of the next. Females lay a single egg at approximately weekly intervals during the wet season; egg production is much reduced during the dry season [17,18]. Population density reaches an annual maximum by the end of the wet season as a result of wet season recruitment [18]. During the wet season juveniles are most abundant in July-August (hatched from eggs laid at the beginning of the wet season—May), fewer juveniles are present later in the wet, suggesting that recruitment declines as the wet season progresses [19]. Annual fluctuations in abundance are most likely due to survivorship of eggs, rather than adult food intake or condition, and abundance is not limited by food availability [18,20]. With respect to climate, lower wet season rainfall and a longer wet season (shorter dry season) are positively associated with lizard abundance [21]. The length of the egg laying period is extended when the wet season is longer, thus increasing the time for egg production and female fecundity [22]. In contrast, increased rainfall during the wet season is associated with greater egg mortality by *Solenopsis* ants [23]. Indicating, that the distribution of rainfall across the year is important to abundance, not necessarily just absolute amounts.

**Thermoregulation.** Field body temperatures (T_b_) range from 24–29°C in the dry season and 25–31°C in the wet season [24]. The difference in T_b_ is due to lizards actively thermoregulating and maintaining a higher T_b_ in the wet season, despite cooler air temperatures [24]. Field T_b_ in the wet season when the lizards were not water stressed was 27.8°C, and is used here as a proxy for preferred T_b_ [24]. Data on thermal optimum is not available for this species.

**Censuses.** Details of census protocols are described elsewhere [21]. In brief, censusing was conducted in December of each year (or January the following year) to correspond to the time of maximum population density at two sites: AVA (A.V. Armour trail, 9°08’33”, -79°51’27”) and Lutz (9°08’39”, -79°50’13”). Mark-recapture studies were used at Lutz site to monitor population density (Lincoln-Peterson estimates of absolute population density) from 1971–1982 and in 1990 and 1994 (Andrews, unpublished). To facilitate censusing more sites, the general censusing protocol was changed in 1983 such that population estimates were relative rather than absolute values (Andrews 1991). Briefly, relative density censuses were conducted by two persons who visually searched for lizards as they walked flagged transects through the study areas. The time spent searching was distinguished from the time spent capturing and processing lizards and density was expressed as the number of *A*. *limifrons* captured per person hour of search, that is, relative density. Areas searched were 30 X 32 m (960m^2^). Relative density censuses were conducted annually from 1983–1999/2007 at Lutz and AVA sites and the mean of these two sites were used in these analyses. Exceptions were: AVA was not censused in 1987; the estimate used in analyses was the mean of the density estimates for 1986 and 1988, and in 1994 when multiple censuses were conducted the site means of these multiple estimates of population density were used. All sighted lizards were caught and their sex, snout vent length (SVL) and weight were recorded. Research was approved by the Smithsonian Tropical Research Institute (STRI) and the STRI Institutional Animal Care and Use Committee.

**Abundance, population growth rate and annual recruitment.** Population growth rate is the log change in abundance between subsequent years (PGR = log(N_t+1_/N_t_). To investigate how climate affects recruitment at different times of the year we subdivided the population into three cohorts; juveniles, young and adults. Lizards were allocated to these cohorts based on snout vent length (SVL); i) juveniles (SVL<35mm, <50days) were lizards that hatched November from eggs laid in September; ii) young (SVL = 35–43mm, <140days) were lizards that hatched in September from eggs laid in July; and iii) adults (SVL>43mm, >140days) were lizards that hatched in July from eggs laid in May. In analyses we use the total number of lizards in each of these cohorts. For more detail see S1 Supporting Information.

### Climate data analysis

Climate variables were calculated from temperature and rainfall data from BCI provided by the Terrestrial-Environmental Sciences Program of the Smithsonian Tropical Research Institute (http://stri.si.edu/sites/esp/). We used climate indices defined by Aguilar et al [3]. For definitions and details see S2 Supporting Information and S2, S3 Tables and S3–S6 Figs.

### Modelling procedure

To investigate cycles in abundance and population growth rate we used autocorrelation and partial autocorrelation analyses (See S3 Supporting Information). Abundance was related to the previous years’ abundance (S3 Supporting Information), therefore in subsequent models we included an autoregressive (AR) term in models of abundance. We also investigated cross correlations between abundance/population growth rate and climate variables (S3 Supporting Information). We found that for SOI, minimum temperature and wet season rainfall (WSR) the previous years’ (*x*_*t-1*_) value was more strongly correlated with abundance or PGR than the current year (*x*_*t*_). As a result we include previous years’ SOI, minimum temperature and wet season rainfall in our models.

To investigate how climate relates to lizard abundance at BCI we used the information theoretic (IT) model selection approach based on Akiake’s Information Criterion (AIC)[25]. A detailed description and justification of the modelling procedure is provided in the S3 Supporting Information. In brief, we used Akiake weights to distinguish between models. An Akiake weight close to 1 is good evidence of a single best model, however if models are poor, then model weights will be low and several models can share similarly low probabilities; suggesting model selection uncertainty. For the analysis of total abundance we included an autoregressive term in all models to account for the dependence of total abundance on abundance in the previous year (See S3 Supporting Information). For models of abundance we fit only a single climate variable to each model to avoid problems of overfitting (>3 parameters). The candidate set of models included climate variables previously associated with abundance, variables that have changed since 1971, SOI, previous years’ values of SOI, minimum temperature and WSR, the number of days that the maximum temperature exceeds *A*. *apletophallus’* field-preferred body temperature, maximum dry and wet season temperature. Refer to Table 1 for a list of variables and justification for their inclusion. For population growth rate we considered the same climate variables, however we did not include an AR term in the model and we considered multiple climate variables and their interactions in a multiple regression framework. To avoid problems of collinearity we only included one rainfall and one temperature variable in multiple regression models. To model how climate affects each cohort we considered the weather conditions pertinent to that cohort (Juveniles = September-December, Young = July-December, and Adults = May-December). We fit simple and multiple regression models, and only included one temperature and one rainfall variable in multiple regression models. For all four response variables we included in the candidate set of models a model with a dummy variable and the minimal model with no climate variable (AR or intercept only). Prior to analysis all the variables were checked for normality, transformed where necessary, centred and standardised. All analyses were carried out using custom scripts in R [26].

**Table 1:** Explanatory variables used in the model set and justification of their inclusion.

Model	Reasoning
Annual Rainfall^1^. Total annual precipitation (ppt)	Abundance related to annual rainfall [21]
Wet Season Length (WSL). Sum of ppt in December_*i*_ and April_*i-1*_	Longer wet season greater egg production [21]
Minimum temperature (Tmin) ^1^. Annual minimum value of temperature	Minimum temperature has increased over time*
Maximum temperature (Tmax) ^1^. Annual maximum value of temperature	Maximum temperature has decreased over time*
Southern oscillation index (SOI). Pressure differential between Darwin and Tahiti	SOI related to rainfall and temperature [3]
Wet season rainfall (WSR). Sum of ppt during June, July and August	Wet season rainfall negatively related to abundance [23]
Number of days the maximum temperature is above *A*. *apletophallus* field-preferred body temperature (PBT) of 28.7 (Tmax>PBT)^1^	Number of days exceeding PBT causes thermoregulatory stress, which will negatively effect population growth rate and recruitment [12]
Mean maximum dry season temperature (MDT): Mean Tmax for January, February and March	Maximum dry season temperature limits population growth rate and recruitment [12]
Mean maximum wet season temperature (MWT): Mean Tmax for June, July and August	Maximum wet season temperature limits population growth rate and recruitment [12]

* Results presented in S1 and S2 Tables.

^1^ These climate variables were also calculated for May-December, July-December and September-December for the analysis of the three different cohorts.

## Results

### Changes in lizard total and cohort-specific abundance

**Fig 1:**
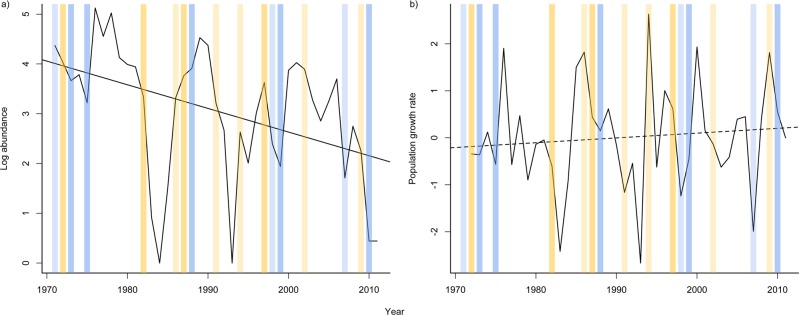
Temporal trends in a) log abundance and b) population growth rate of *Anolis apletophallus* surveyed in an annual December census for 40 years. Yellow shaded bars show strong (darker shading) and moderate (lighter shading) *el nino* events and blue bars show strong (darker shading) and moderate (lighter shading) *la nina* events (see http://ggweather.com/enso/oni.htm for description of strength ranking).

**Fig 2:**
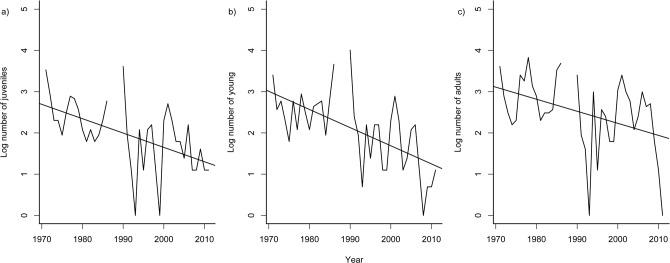
Temporal trends in a) log number of juveniles, b) log number of young, and c) log number of adults of *Anolis apletophallus* surveyed in an annual December census for 40 years.

Overall abundance of *A*. *apletophallus* on BCI has declined over the last 40 years (Fig. 1, *F*_1,39_ = 8.13, *p* = 0.005, r^2^ = 0.16, rate of decline ∼ 1.08 lizards/1km^2^/yr) and across all cohorts (juveniles: *F*_1,36_ = 14.68, *p* < 0.001, r^2^ = 0. 26; young: *F*_1,36_ = 22.43, *p* < 0.001, r^2^ = 0. 36, and adults *F*_1,36_ = 7.05, *p* = 0.011, r^2^ = 0. 14) (Fig. 2). Population growth rate has not changed in the last 40 years (Fig. 1, *F*_1,38_ = 0.42, *p* = 0.52, r^2^ = -0.05). Total abundance was positively correlated with abundance of all three cohorts (juveniles/young/adults, S1 Fig.). Population growth rate was positively related to the log number of adults and juveniles, but not to the log number of young (S1 Fig.). Log abundance was positively correlated with population growth rate (S2 Fig., *F*_1,38_ = 5.18, *p* = 0.02, r^2^ = 0.09), an expected trend for a declining population.

### Temporal trends and cycles

Although large fluctuations in abundance were observed across the 40-years, there was little evidence of significant long-term cycles in abundance. Auto- and partial autocorrelation analysis identified 1-year lag in abundance, i.e. abundance in any year was positively correlated to abundance in the previous year (S3 Supporting Information, S8 Fig.). In contrast, a 7-year cycle in population growth rate was observed (S8 Fig.), population growth rate at time *t0* was correlated with population growth rate *t7* and *t15*.

### Local climate changes over time

Since 1971, rainfall intensity, very wet days, extremely wet days, and minimum temperature have increased at BCI, whereas diurnal temperature range, maximum temperature, percentage of cool nights, and maximum wet season temperature have decreased (S2 Table, S3, S4 Figs.). This pattern is consistent with overall trends for the region with the exception that diurnal temperature range and maximum temperature have increased on a regional scale [3] but have decreased at BCI. Climatic variables positively related to the southern oscillation index (SOI) were rainfall intensity, very wet days, and percentage of cool nights and negatively related to SOI were minimum temperature and percentage of warm nights (S2 Table, S5, S6 Figs.).

### Abundance relationships with climate

**Fig 3:**
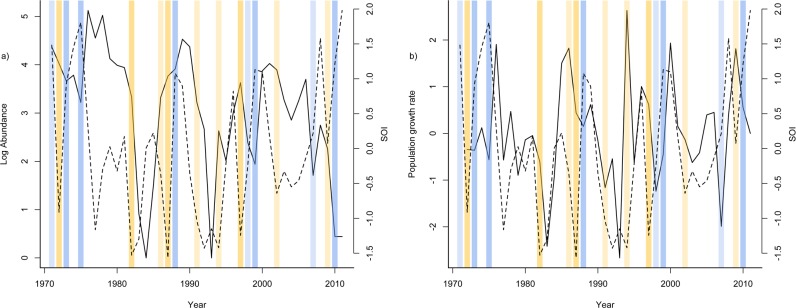
Temporal trends in a) log abundance (solid line) and southern oscillation index (SOI) (dashed line) and b) population growth rate (solid line) with SOI (dashed line) on BCI over the last 40 years. Yellow shaded bars show strong (darker shading) and moderate (lighter shading) *el nino* events and blue bars show strong (darker shading) and moderate (lighter shading) *la nina* events (see http://ggweather.com/enso/oni.htm for description of strength ranking).

The first set of models investigated how total lizard abundance and climate variables were related. Of the candidate set of models, Akaike weights support a confidence set of 8 (Table 2). The AIC top 3 models had weights of 0.28, 0.23, and 0.16, suggesting no single climate variable was strongly associated with abundance. The three best models contained the variables SOI_*t-1*_ (Fig. 3), rainfall and minimum temperature. The second set of models investigated how population growth rate was related to climate (Table 3). The Akiake best model with the variable SOI_*t-1*_ had a weight of 0.51, over 3 times as high as the second best model weight, suggesting that population growth rate was positively related to SOI_*t-1*_ (Fig. 3). In the third set of models we investigated the relationship between recruitment of specific cohorts (juveniles, young, and adults) and the climate they experienced. Two models were retained in the confidence set for log abundance of juveniles and young; the additive model containing rainfall and minimum temperature, and the model with rainfall, minimum temperature and their interaction (Tables 4 and 5). The relationships with rainfall and minimum temperature were negative. The results were similar for the log abundance of adults, except a third model was also retained; the model with rainfall only (Table 6). For all cohorts the Akiake weight of the best model was substantially higher than the second best model, indicating that this model out performed all the other models we considered. In all cases the additive model out performed the model with the interaction between rainfall and minimum temperature, and the slope estimate and confidence intervals for the interaction overlapped zero. This strongly suggests that there was no effect of an interaction between rainfall and minimum temperature on cohort specific abundance. Taken together these analyses suggest that population growth rate was positively related to the previous year SOI (*la niña* phases of the SOI) and yearly recruitment is negatively related to increasing rainfall and minimum temperature.

**Table 2:** Summary statistics from linear models retained in the confidence set (evidence ratio >0.13) for log abundance.

Variable	K	AICc	Δi	ωi	r2	σ2	Slope Estimate (95% CI)
SOI_*t-1*_	3	99.91	0.00	0.28	0.56	0.92	0.36 (0.03, 0.69)
Rainfall	3	100.30	0.39	0.23	0.55	0.93	-0.33 (-0.64, -0.02)
Tmin	3	101.00	1.09	0.16	0.54	0.94	-0.31 (-0.64, 0.01)
Tmin_*t-1*_	3	101.78	1.87	0.11	0.48	0.95	-0.41 (-0.76, 0.07)
MWT	3	102.89	2.98	0.06	0.52	0.96	0.27 (-0.12, 0.66)
Tmax>PBT	3	102.93	3.03	0.06	0.52	0.96	0.25 (-0.11, 0.61)
WSR	3	103.24	3.33	0.05	0.51	0.97	-0.22 (-0.56, 0.13)
Tmax	3	103.63	3.72	0.04	0.51	0.97	0.22 (-0.17, 0.62)

*K* = number of model parameters, AICc = corrected AIC, Δ_i_ difference in model AIC and the AIC best model (AIC_min_), (ω_i_) = AIC weight, r^2^ = regression coefficient, σ^2^ = model residual variance.

Model parameters for model without a climate variable: Auto Regressive term only Δ_I_ = 18.98, σ^2^ = 1.01.

**Table 3:** Summary statistics from linear models retained in the confidence set (evidence ratio >0.13) for population growth rate.

Variable	K	AICc	Δi	ωi	r2	σ2	Slope Estimate (95% CI)
SOI _*t-1*_	2	106.90	0.00	0.51	0.13	1.04	0.40 (0.03, 0.77)
Tmin	2	109.19	2.29	0.16	0.07	1.08	-0.28 (-0.65 -0.09)
Rainfall							-0.25 (-0.59, -0.10)
+Tmin	3	109.61	2.70	0.13	0.12	1.06	-0.29 (-0.57, 0.07)
Rainfall	2	109.88	2.98	0.11	0.05	1.09	-0.23 (-0.58, 0.13)
SOI	2	110.45	2.29	0.09	0.03	1.10	0.21 (-0.19 -0.60)

*K* = number of model parameters, AICc = corrected AIC, Δ_i_ difference in model AIC and the AIC best model (AIC_min_), (ω_i_) = AIC weight, r^2^ = regression coefficient, σ^2^ = model residual variance.

Model parameters for model without a climate variable: intercept only Δ_I_ = 20.27, σ^2^ = 1.14.

**Table 4:** Summary statistics from linear models retained in the confidence set (evidence ratio >0.13) for log number of juveniles (>35mm) and climate variables (September-December).

Variable	K	AICc	Δi	ωi	r2	σ2	Slope Estimate (95% CI)
Rainfall							-0.43 (-0.65, -0.21)
+Tmin	3	75.81	0.00	0.79	0.45	0.62	-0.44 (-0.66 -0.22)
Rainfall							-0.43 (-0.65, -0.21)
+Tmin							-0.45 (-0.67, -0.23)
+Rainfall:Tmin	4	78.48	2.67	0.21	0.45	0.63	-0.03 (-0.25, 0.19)

*K* = number of model parameters, AICc = corrected AIC, Δ_i_ difference in model AIC and the AIC best model (AIC_min_), (ω_i_) = AIC weight, r^2^ = regression coefficient, σ^2^ = model residual variance.

Model parameters for model without a climate variable: intercept only Δ_I_ = 18.69, σ^2^ = 0.80.

**Table 5:** Summary statistics from linear models retained in the confidence set (evidence ratio >0.13) for log number of young (36–43mm) and climate variables (July-September).

Variable	K	AICc	Δi	ωi	r2	σ2	Slope Estimate (95% CI)
Rainfall							-0.40 (-0.65, -0.21)
+Tmin	3	87.42	0.00	0.76	0.37	0.72	-0.42 (-0.66 -0.22)
Rainfall							-0.40 (-0.66, -0.14)
+Tmin							-0.42 (-0.66, -0.18)
+Rainfall:Tmin	4	90.18	2.76	0.19	0.37	0.74	-0.01 (-0.25, 0.23)

*K* = number of model parameters, AICc = corrected AIC, Δ_i_ difference in model AIC and the AIC best model (AIC_min_), (ω_i_) = AIC weight, r^2^ = regression coefficient, σ^2^ = model residual variance.

Model parameters for model without a climate variable: intercept only Δ_I_ = 11.61, σ^2^ = 0.84.

**Table 6:** Summary statistics from linear models retained in the confidence set (evidence ratio >0.13) for log number of adults (≥44mm) and climate variables (May-December).

Variable	K	AICc	Δi	ωi	r2	σ2	Slope Estimate (95% CI)
Rainfall							-0.45 (-0.65, -0.21)
+Tmin	3	92.38	0.00	0.68	0.34	0.75	-0.28 (-0.66 -0.22)
Rainfall							-0.40 (-0.66, -0.18)
+Tmin							-0.28 (-0.52, -0.04)
Rainfall:Tmin	4	95.05	2.68	0.18	0.34	0.76	-0.03 (-0.21, 0.27)
Rainfall	2	95.60	3.22	0.14	0.23	0.80	-0.42 (-0.68, -0.16)

*K* = number of model parameters, AICc = corrected AIC, Δ_i_ difference in model AIC and the AIC best model (AIC_min_), (ω_i_) = AIC weight, r^2^ = regression coefficient, σ^2^ = model residual variance.

Model parameters for model without a climate variable: intercept only Δ_I_ = 10.56, σ^2^ = 0.90.

## Discussion

The aim of this study was to identify temporal trends in lizard populations and climate on BCI, and investigate how climate was related to lizard abundance, population growth rate and annual recruitment. We identified changes in climate and a dramatic decrease in lizard abundance over the last 40 years on BCI. Mean abundance during 1970–1980 was ∼70 lizards per 960m^2^, in the last decade this has fallen to ∼25 lizards/960m^2^. Population growth rate was positively related to the SOI of the previous year. Positive SOI represents the *la niña* phase of the southern oscillation and brings generally cooler and wetter conditions (S2 Supporting Information) [3,27]. Annual recruitment, as measured as cohort specific abundance, was negatively related to increasing rainfall and minimum temperatures experienced during that year. Recent analyses of SOI have demonstrated increases in variance over the last 30 years [28] and severe *el nino* events are predicted to increase in the future with climate change [29]. These predicted changes in SOI could have negative consequences for this species’ future on BCI.

Our data suggest that SOI phases may be driving changes in population growth rate of lizards on BCI, but does this contribute to the decline in abundance we observed? Positive SOI, or *la niña*, periods were followed by higher abundance and population growth rate in the following year. Several population crashes followed severe *el niño* events (1982–3, 1991–2, 2009–10), and lizard abundance was high during prolonged *la niña* phases (1970s, 1998–2000). Our study did not identify a directional temporal trend in SOI, but more robust methods to detect temporal changes in SOI have demonstrated that the period 1979–2009 had much greater variability in SOI *el nino* than the previous ∼400 years [28]. Extreme *el nino* events are also expected to increase in frequency with global warming [29]. It is possible then that changes in SOI variability, in particular more severe *el nino* events experienced in the last 30 years may be responsible for the marked population decline we observed on BCI. Previous studies have demonstrated wide-ranging affects of SOI on the plants and mammals of BCI [27,30], and our results demonstrate that these global climate cycles are also affecting the *A*. *apletophallus* population.

Our results suggest that *la niña* phases, which bring lower minimum temperatures, will have a positive effect on annual recruitment (Table 3), however changes in population growth rate are not observed until the following year. One explanation for this lag may be that November-December eggs and hatchlings, which are not included in the census, could make an important contribution to the following years’ population growth. The eggs are not surveyed and it is likely that very small hatchlings (<20mm) are overlooked in the census; they only make up about 1% of all lizards caught. These eggs and hatchlings will become adults early in the wet season the following year and produce the first eggs for that year. Thus, high recruitment late in the wet season as a result of the favourable climate (*la nina*) conditions during the year may drive increased population growth rate the following year, and could explain the one year lag between SOI and population growth rate.

Rainfall and minimum temperature were negatively related to abundance and annual recruitment (adult, young and juveniles). Again we wish to address the question, are changes in rainfall or minimum temperature driving the decline in abundance we observed. A negative effect of rainfall on abundance is consistent with the results of a previous analyses of the first 19 years of this data [21]. A feature of this system is that we have a plausible mechanism linking rainfall to abundance. Manipulation studies revealed that high rainfall and soil moisture negatively effect egg survival through increased predation by *Solenopsis* ants [23]. However, it is unclear if rainfall explains the decline in abundance that we have observed on BCI, because rainfall has not changed significantly during the last 40 years. Although, across the region most weather stations recorded a positive trend in rainfall, including BCI, trends were not significant possibly because of large interannual variation and a relatively short time period [3]. Our ability to detect changes in rainfall over time with this dataset are limited, as a result it is not possible to conclude that changes in rainfall are driving the population decline observed on BCI.

The other climate variable that was negatively related to recruitment and could be related to the decline we observe was minimum temperature. Minimum temperature has increased in the past 40 years; we observed a dramatic increase between 1970–1990 and a levelling off in the last 20 years (S4 Fig.). Increasing minimum temperature equates to higher night-time temperature. We do not know why higher night-time temperature may be negatively related to the lizard recruitment, it may be the result of a direct effect on the lizards and/or their predators, or indirect effects on the forest as a whole. Speculating on direct effects, it is possible that higher night-time temperature is associated with increased energy demands of lizards and/or increased activity of nocturnal ectothermic predators, such as ants, spiders and snakes. Alternatively indirect effects may be impacting the lizards as a result of changes to the forest; Whitfield et al. [13] attribute reduced abundance of amphibians and reptiles at a rainforest preserve in Costa Rica to a climate-related reduction in leaf litter. There is no evidence to suggest there has been a reduction in leaf litter on BCI [31], but there have been other changes to the forest structure over time, i.e. forest succession in younger stands [32] and increased liana density [33]. Testing some of these hypotheses will be imperative if we are to understand why *A*. *apletophallus* has declined and for predicting its persistence in the future.

In this study we document a decline in the abundance of a tropical lizard *A*. *apletophallus*, at BCI using 40 years of abundance data. Unsurprisingly, the local climate has also changed over the study period; rainfall intensity and minimum temperature has increased, while maximum temperature has decreased. In an attempt to understand how climate change is related to lizard abundance, we tested for relationships between population growth rate, abundance and annual recruitment with temperature and rainfall variables. We found evidence that population growth rate was higher following southern oscillation *la niña* years, and within-year recruitment was negatively affected by increasing rainfall and minimum temperature in the same year. We conclude that SOI phases are driving long-term fluctuations in population growth rate, and changes in night-time temperature may be contributing to the population decline observed on BCI.

## Supporting Information

S1 Fig Relationship between log abundance and cohort specific abundance. (PDF)

S2 Fig Relationship between log abundance and population growth rate. (PDF)

S3 Fig Trends in rainfall indices from 1971–2011 at Barro Colorado Island. (PDF)

S4 Fig Trends in temperature indices from 1971–2011 at Barro Colorado Island. (PDF)

S5 Fig Relationship between Southern Oscillation Index and rainfall indices at Barro Colorado Island. (PDF)

S6 Fig Relationship between Southern Oscillation Index and temperature indices at Barro Colorado Island. (PDF)

S7 Fig Correlation matrix of 14 climate variables initially considered for inclusion in modelling. (PDF)

S8 Fig Autocorrelation and partial autocorrelation of abundance and population growth rate (PGR). (PDF)

S9 Fig Temporal trends between log abundance and Southern Oscillation Index, rainfall wet season length and wet season rainfall. (PDF)

S10 Fig Temporal trends between log abundance and minimum temperature, maximum temperature, number days exceeding field-preferred body temperature, maximum dry season temperature and maximum wet season temperature. (PDF)

S11 Fig Temporal trends between population growth rate and southern oscillation index, rainfall, wet season length and wet season rainfall. (PDF)

S12 Fig Temporal trends between population growth rate and minimum temperature, maximum temperature, number days exceeding field-preferred body temperature, maximum dry season temperature and maximum wet season temperature. (PDF)

S13 Fig Temporal covariation of log abundance and population growth rate and cross correlation between population growth rate and abundance. (PDF)

S14 Fig Cross correlation of log abundance and southern oscillation index, rainfall, wet season length and wet season rainfall. (PDF)

S15 Fig Cross correlation of log abundance and minimum temperature, maximum temperature, number days exceeding field-preferred body temperature, maximum dry season temperature and maximum wet season temperature. (PDF)

S16 Fig Cross correlation of population growth rate and southern oscillation index, rainfall, wet season length and wet season rainfall. (PDF)

S17 Fig Cross correlation of population growth rate and minimum temperature, maximum temperature, number days exceeding preferred body temperature, maximum dry season temperature and maximum wet season temperature. (PDF)

S18 Fig Principle component biplot of climate variables and log abundance. (PDF)

S19 Fig Principle component biplot of climate variables and population growth rate. (PDF)

S1 Supporting Information Abundance, population growth rate and cohort specific abundance. Details of how measures of abundance, population growth rate and cohort specific abundance (recruitment) were estimated, and are correlated.
(PDF)

S2 Supporting Information Climate Data. Description of the climate indices and an analysis of how these have changed over time and how these are correlated.
(PDF)

S3 Supporting Information Data Analysis. Detailed description of the cross-correlation analysis and information theoretic modelling procedure
(PDF)

S1 Table Description of climate change indices used in the study. (PDF)

S2 Table Results table of linear relationships between climate change indices and time from 1971–2011. (PDF)

S3 Table Relationship between Southern Oscillation Index (SOI) and other climate variables from 1971–2011. (PDF)

S4 Table Table of mean abundance, number of young, number of juveniles and number of adults from 1971–2011. (PDF)
