# Supplementary material for: Long-Term Data Reveal a Population Decline of the Tropical Lizard Anolis apletophallus, and a Negative Affect of El Nino Years on Population Growth Rate
Source: PLoS One. 2015 Feb 11;10(2):e0115450. doi: 10.1371/journal.pone.0115450 (PMC4325001; doi:10.1371/journal.pone.0115450)

Figure S1. Relationship between log abundance and cohort specific abundance. Log abundance was positively related to cohort specific abundance. Points and fitted (line) relationships of log abundance and the number of a) adults, b) young and c) juveniles. Population growth rate was positively related to log number of adults and juveniles. Points and significant fitted relationships (line) of population growth rate and log number of d) adults e) young, and f) juveniles.

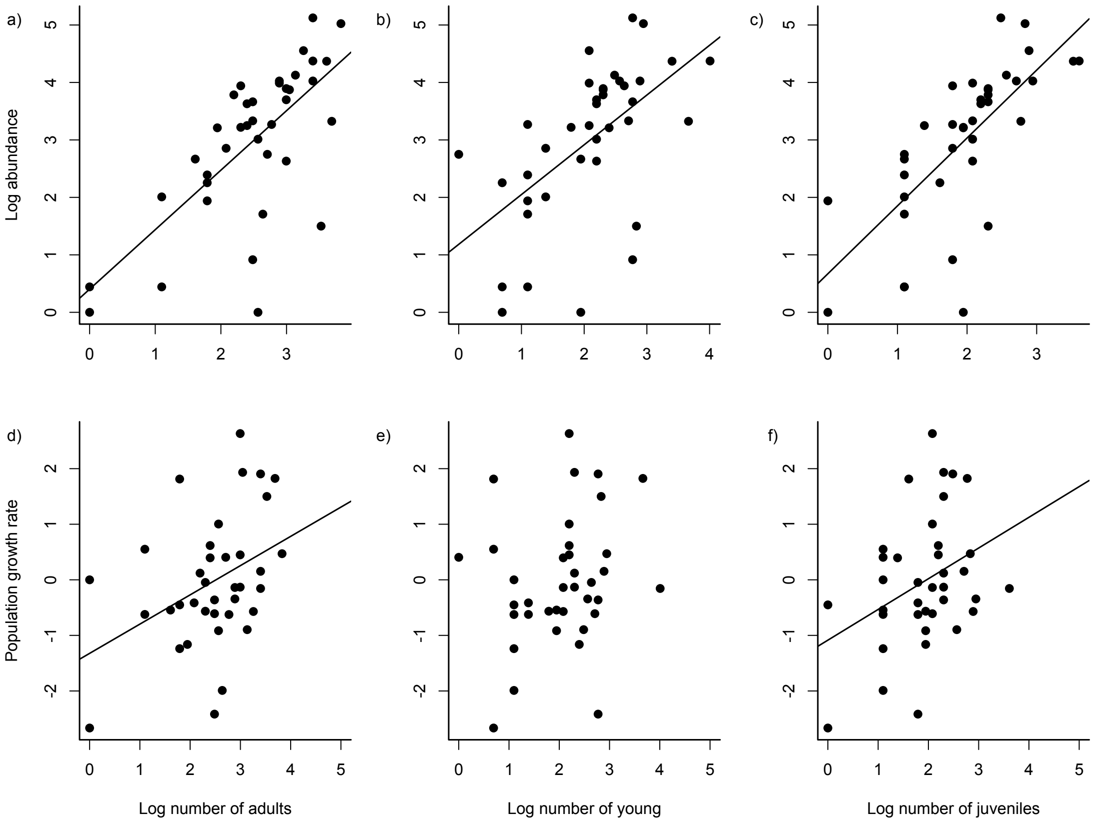

Supplement: S1 Fig — (PDF) [file pone.0115450.s001.pdf]
