# Supplementary material for: Long-Term Data Reveal a Population Decline of the Tropical Lizard Anolis apletophallus, and a Negative Affect of El Nino Years on Population Growth Rate
Source: PLoS One. 2015 Feb 11;10(2):e0115450. doi: 10.1371/journal.pone.0115450 (PMC4325001; doi:10.1371/journal.pone.0115450)

Figure S7. Correlation matrix of 14 climate variables initially considered for inclusion in modelling. On the lower diagonal scatterplot with red trend lines, on the upper diagonal correlation coefficients and asterix indcate significance levels (*<0.05, **<0.01, ***<0.001). Plot made using R Package 'PerfomanceAnalytics', function 'chart.Correlation'.

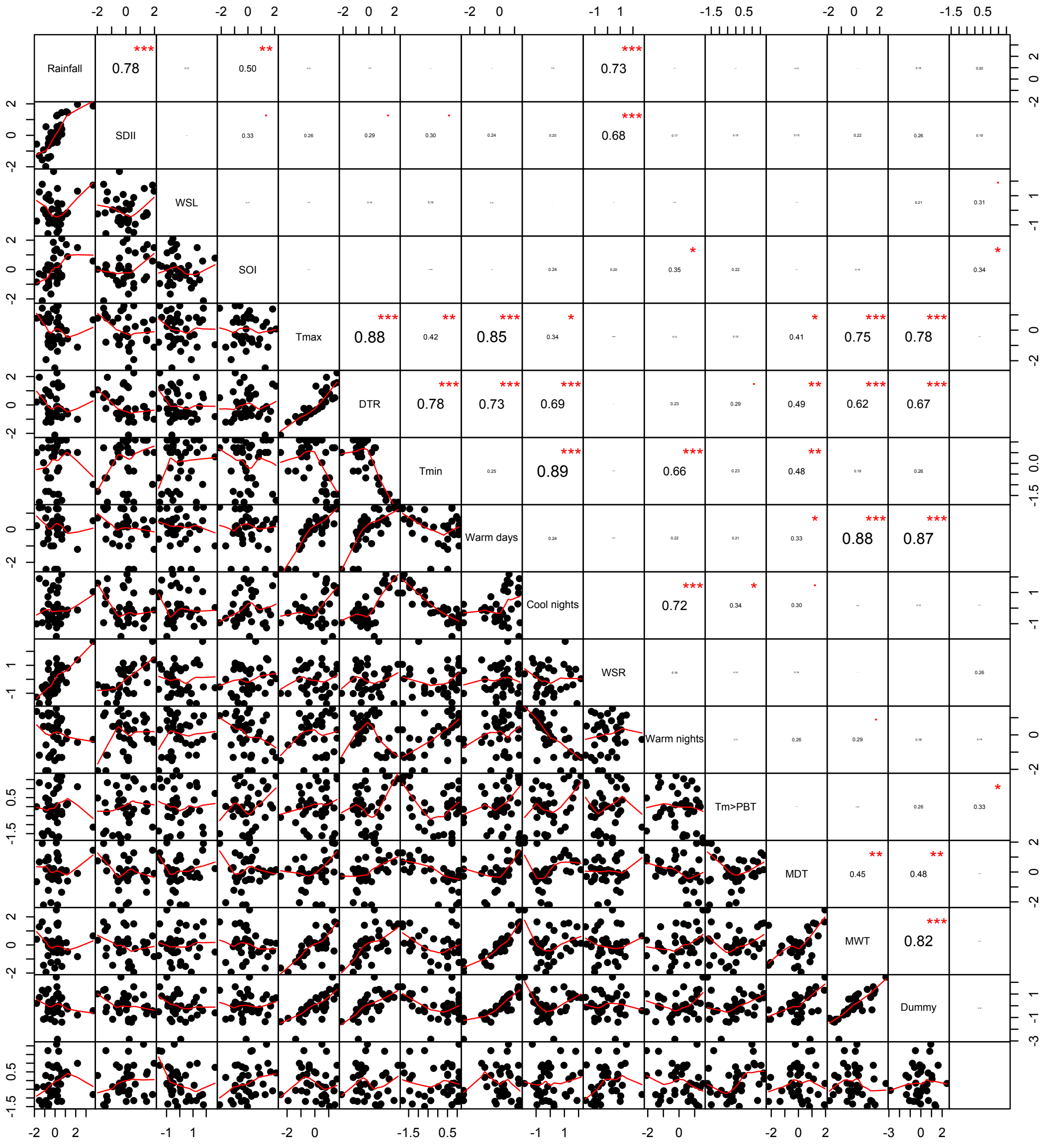

Supplement: S7 Fig — (PDF) [file pone.0115450.s007.pdf]
