# Supplementary material for: Long-Term Data Reveal a Population Decline of the Tropical Lizard Anolis apletophallus, and a Negative Affect of El Nino Years on Population Growth Rate
Source: PLoS One. 2015 Feb 11;10(2):e0115450. doi: 10.1371/journal.pone.0115450 (PMC4325001; doi:10.1371/journal.pone.0115450)

Figure S8. Autocorrelation and partial autocorrelation of abundance and population growth rate (PGR).

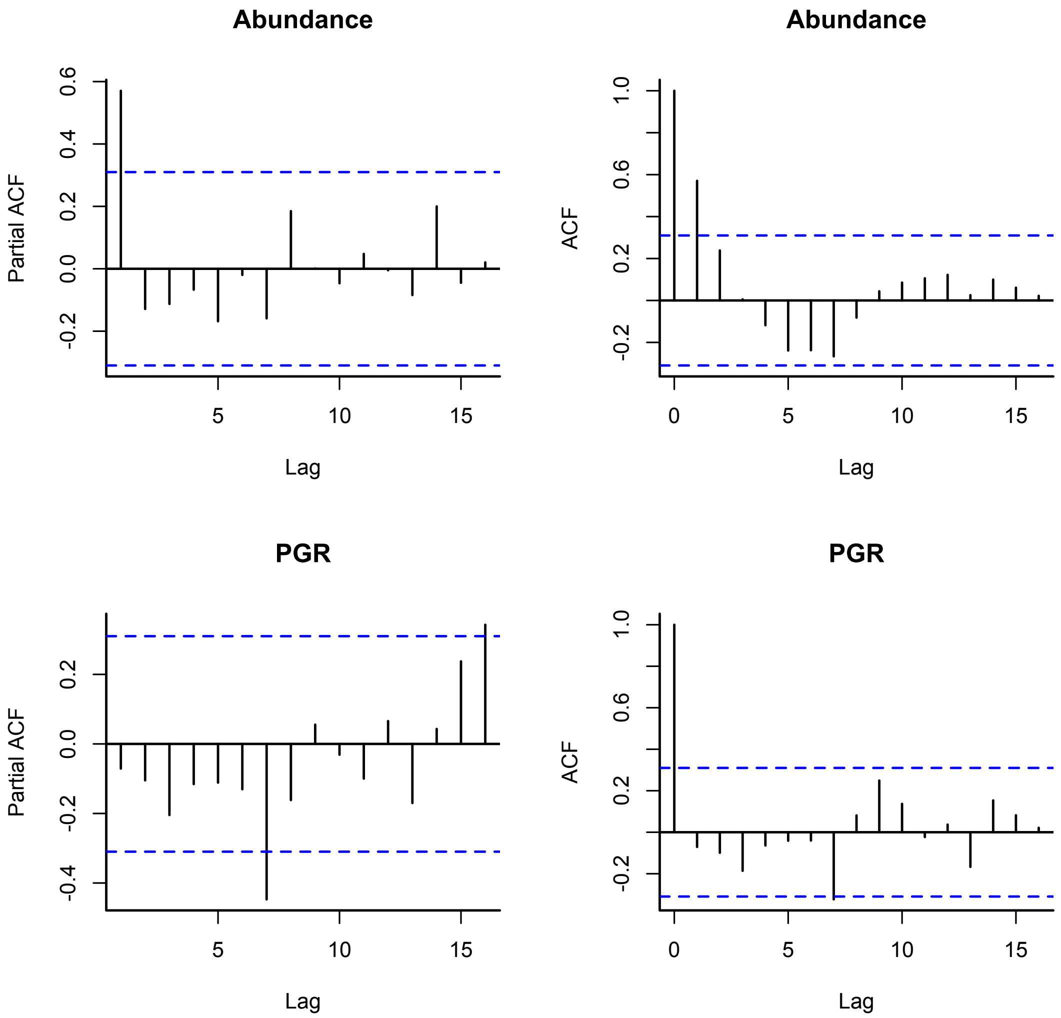

Supplement: S8 Fig — (PDF) [file pone.0115450.s008.pdf]
