# Supplementary material for: Long-Term Data Reveal a Population Decline of the Tropical Lizard Anolis apletophallus, and a Negative Affect of El Nino Years on Population Growth Rate
Source: PLoS One. 2015 Feb 11;10(2):e0115450. doi: 10.1371/journal.pone.0115450 (PMC4325001; doi:10.1371/journal.pone.0115450)

Figure S9. Temporal trends between log abundance and Southern Oscillation Index, rainfall wet season length and wet season rainfall. Southern oscillation index (SOI), rainfall (PPT), wet season length (WSL) and wet season rainfall (WSL).

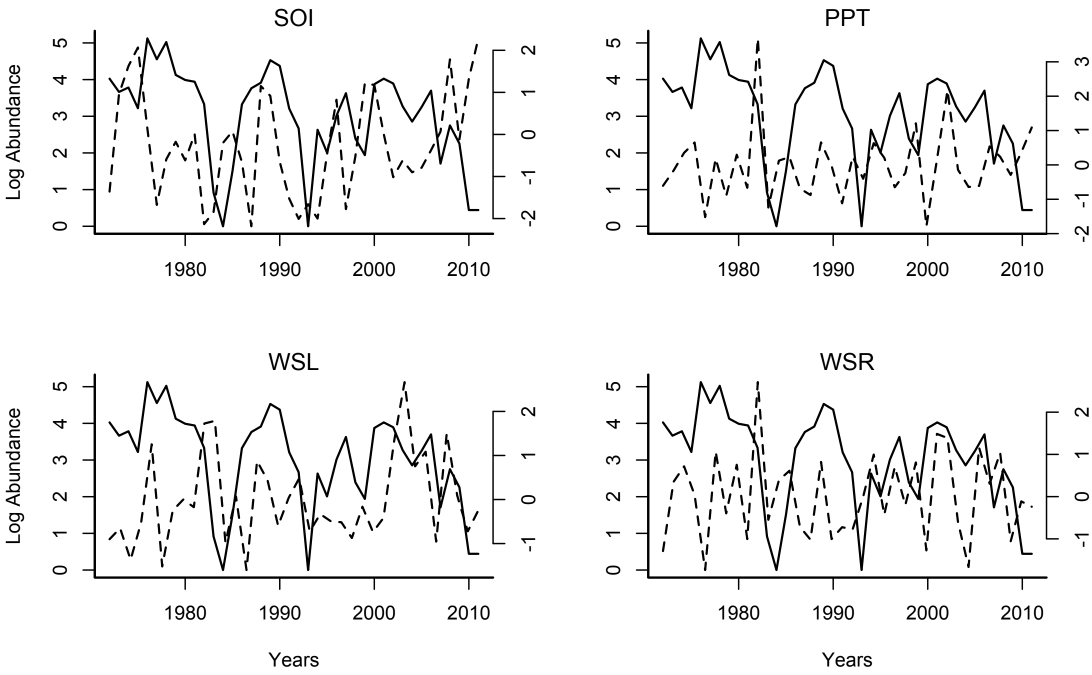

Supplement: S9 Fig — (PDF) [file pone.0115450.s009.pdf]
