# Supplementary material for: Long-Term Data Reveal a Population Decline of the Tropical Lizard Anolis apletophallus, and a Negative Affect of El Nino Years on Population Growth Rate
Source: PLoS One. 2015 Feb 11;10(2):e0115450. doi: 10.1371/journal.pone.0115450 (PMC4325001; doi:10.1371/journal.pone.0115450)

Figure S12. Temporal trends between population growth rate and minimum temperature, maximum temperature, number days exceeding field-preferred body temperature, maximum dry season temperature and maximum wet season temperature. Minimum temperature (Tmin), maximum temperature (Tmax), number days exceeding preferred body temperature (Tmax>PBT), maximum dry season temperature (MDT) and maximum wet season temperature (MWT).

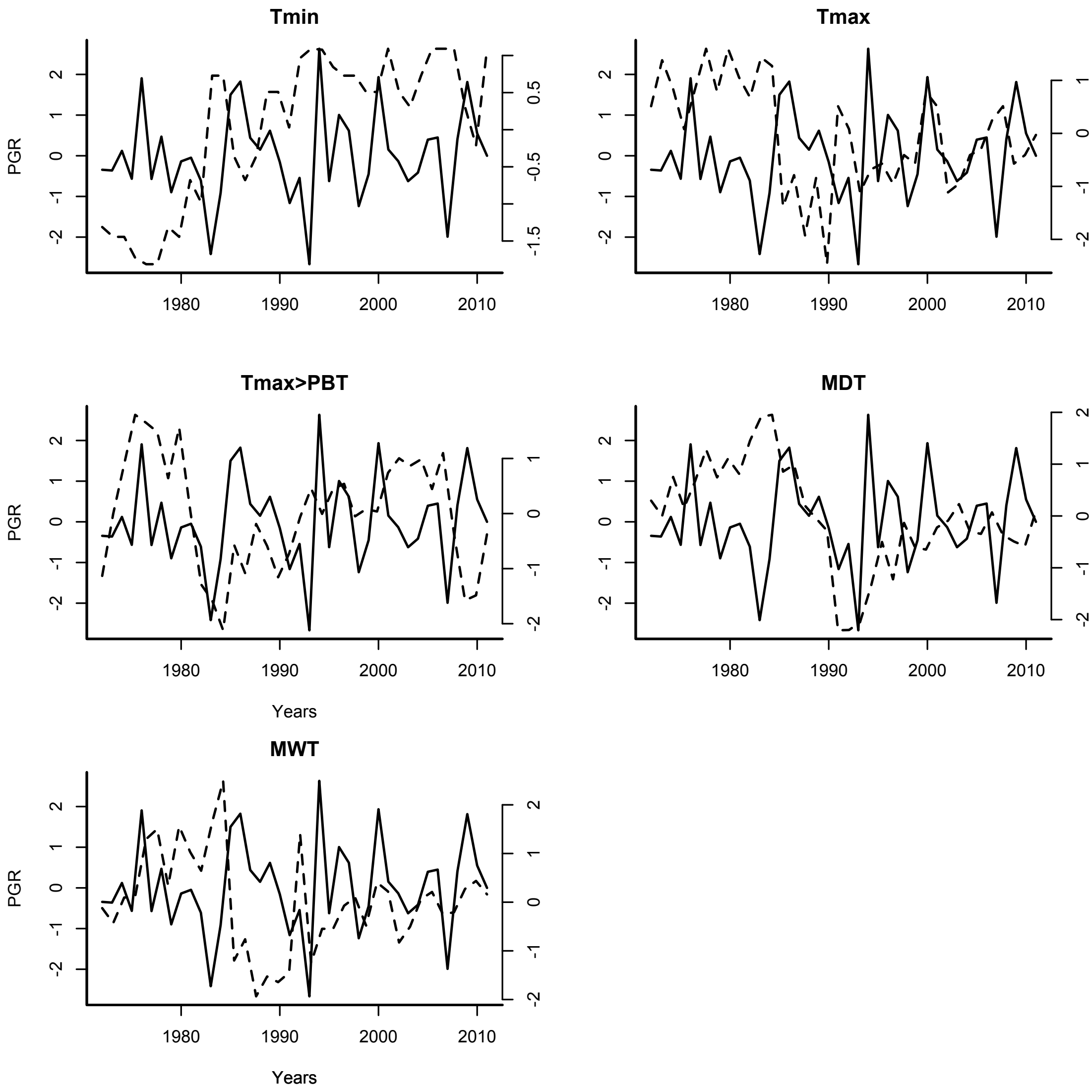

Supplement: S12 Fig — (PDF) [file pone.0115450.s012.pdf]
