# Supplementary material for: Long-Term Data Reveal a Population Decline of the Tropical Lizard Anolis apletophallus, and a Negative Affect of El Nino Years on Population Growth Rate
Source: PLoS One. 2015 Feb 11;10(2):e0115450. doi: 10.1371/journal.pone.0115450 (PMC4325001; doi:10.1371/journal.pone.0115450)

Figure S13. Temporal covariation of log abundance and population growth rate and cross correlation between population growth rate and abundance. Population growth rate (PGR) (upper panel) and cross correlation between population growth rate and abundance (lower panel).

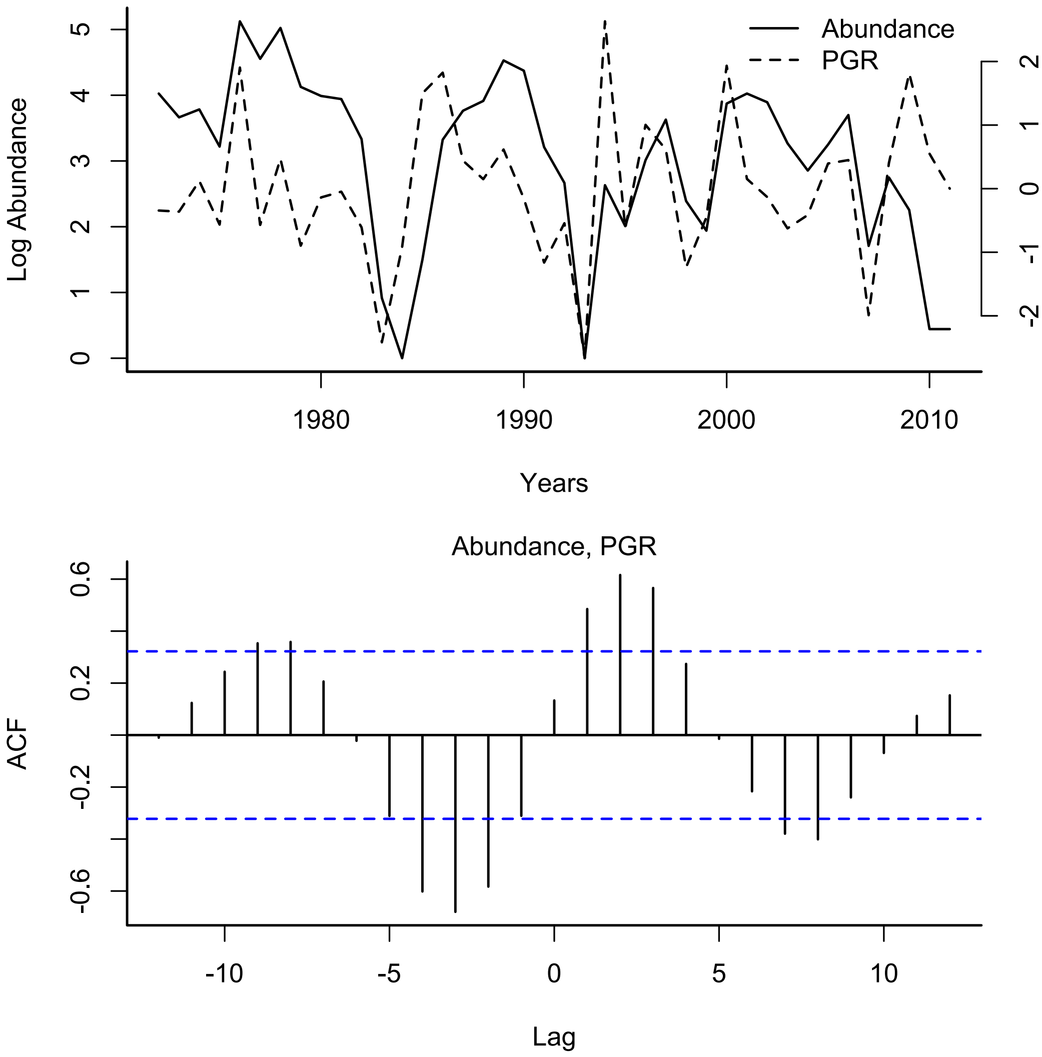

Supplement: S13 Fig — (PDF) [file pone.0115450.s013.pdf]
