# Supplementary material for: Long-Term Data Reveal a Population Decline of the Tropical Lizard Anolis apletophallus, and a Negative Affect of El Nino Years on Population Growth Rate
Source: PLoS One. 2015 Feb 11;10(2):e0115450. doi: 10.1371/journal.pone.0115450 (PMC4325001; doi:10.1371/journal.pone.0115450)

Figure S16. Cross correlation of population growth rate and southern oscillation index, rainfall, wet season length and wet season rainfall. Southern oscillation index (SOI), rainfall (PPT), wet season length (WSL) and wet season rainfall (WSL).

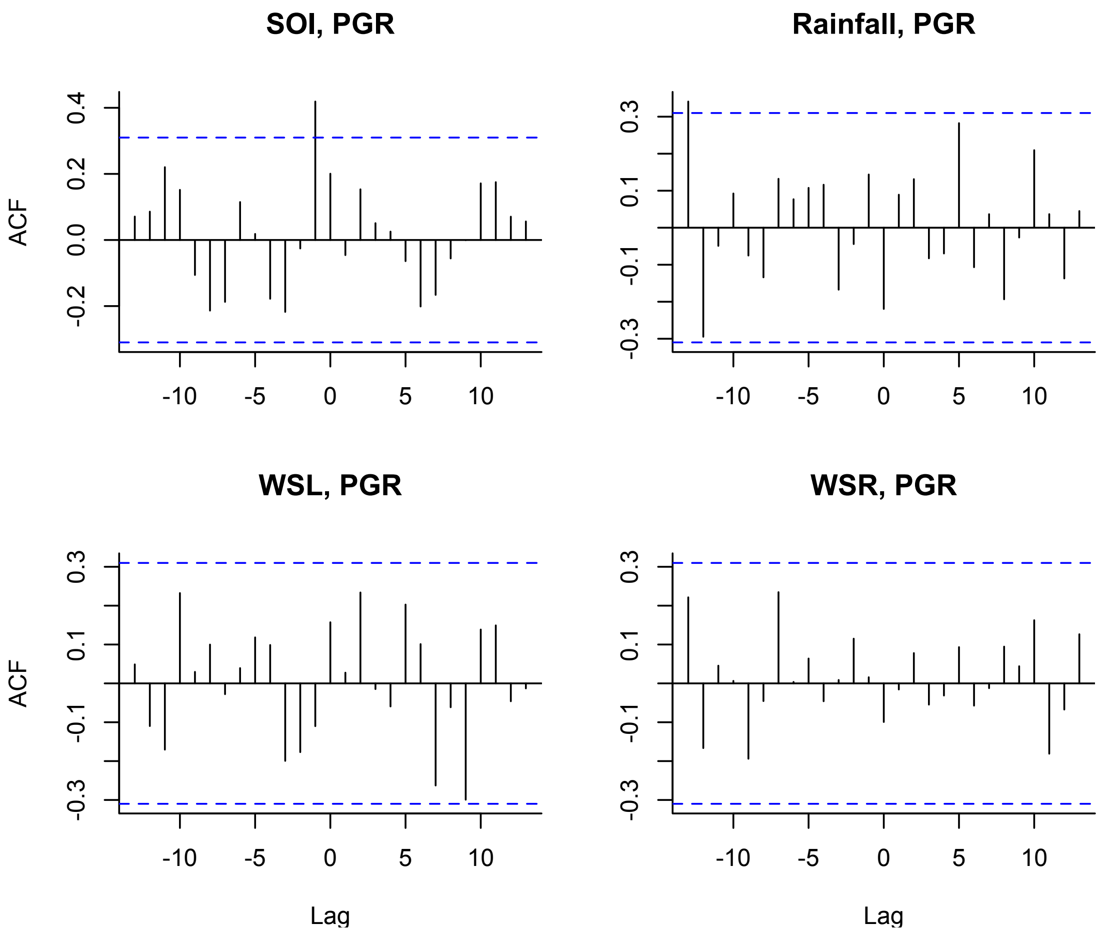

Supplement: S16 Fig — (PDF) [file pone.0115450.s016.pdf]
