# Supplementary material for: Long-Term Data Reveal a Population Decline of the Tropical Lizard Anolis apletophallus, and a Negative Affect of El Nino Years on Population Growth Rate
Source: PLoS One. 2015 Feb 11;10(2):e0115450. doi: 10.1371/journal.pone.0115450 (PMC4325001; doi:10.1371/journal.pone.0115450)

Figure S17. Cross correlation of population growth rate and minimum temperature, maximum temperature, number days exceeding preferred body temperature, maximum dry season temperature and maximum wet season temperature. Minimum temperature (Tmin), maximum temperature (Tmax), number days exceeding preferred body temperature (Tmax>PBT), maximum dry season temperature (MDT) and maximum wet season temperature (MWT).

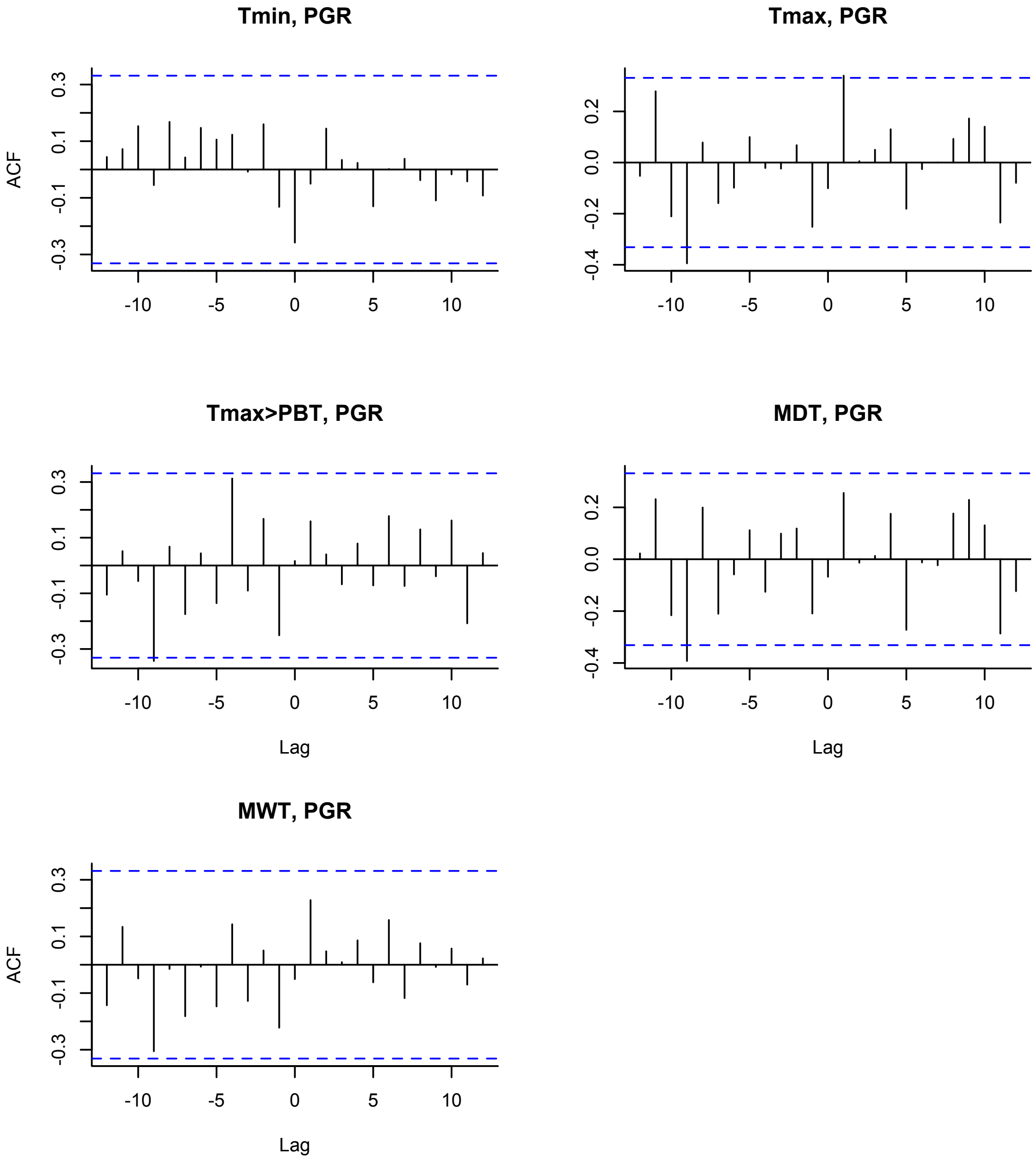

Supplement: S17 Fig — (PDF) [file pone.0115450.s017.pdf]
