# Supplementary material for: Long-Term Data Reveal a Population Decline of the Tropical Lizard Anolis apletophallus, and a Negative Affect of El Nino Years on Population Growth Rate
Source: PLoS One. 2015 Feb 11;10(2):e0115450. doi: 10.1371/journal.pone.0115450 (PMC4325001; doi:10.1371/journal.pone.0115450)

Figure S19. Principle component biplot of climate variables and population growth rate. PGR = Population growth rate, PPT= total annual precipitation, SDII= rainfall intensity, WSR = wet season rainfall, WSL = wet season length, SOI = southern oscillation index, Tmin = minimum temperature, Tmax = maximum temperature, CoolNights = percentage of cool nights, Tmax>PBT = number of days Tmax > preferred body temperature of *A. apletophallus*, MWT = maximum wet season temperature, MDT = maximum dry season temperature. Numbers on biplot indicate years.

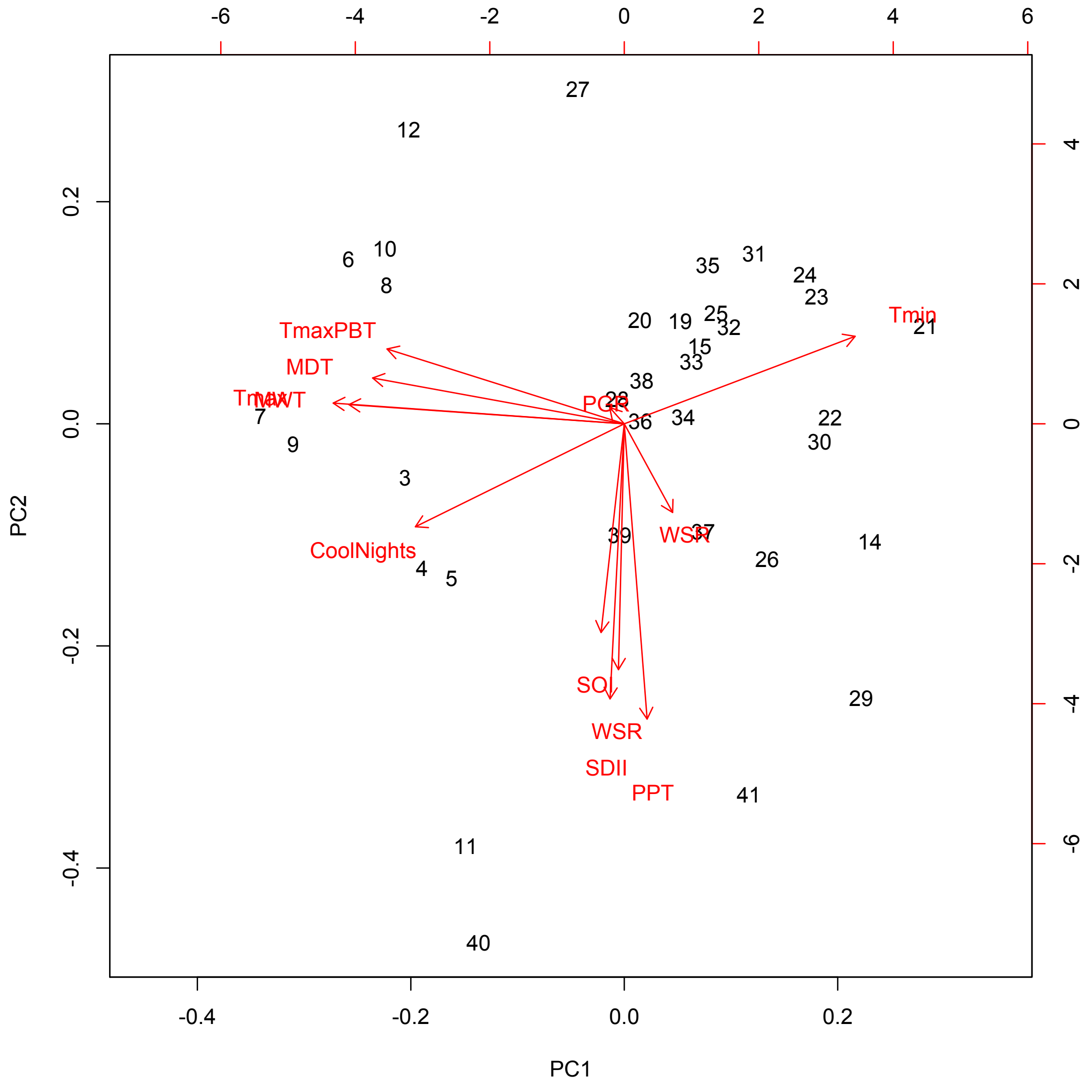

Supplement: S19 Fig — (PDF) [file pone.0115450.s019.pdf]
