# Supplementary material for: Long-Term Data Reveal a Population Decline of the Tropical Lizard Anolis apletophallus, and a Negative Affect of El Nino Years on Population Growth Rate
Source: PLoS One. 2015 Feb 11;10(2):e0115450. doi: 10.1371/journal.pone.0115450 (PMC4325001; doi:10.1371/journal.pone.0115450)
Supplement: S1 Supporting Information — Details of how measures of abundance, population growth rate and cohort specific abundance (recruitment) were estimated, and are correlated. (PDF) [file pone.0115450.s020.pdf]

Supporting Information S1: Abundance, population growth rate and cohort specific abundance.

Abundance data

Abundance is mean relative density (number of *A. apletophallus* captured per person hour of search) across AVA and Lutz sites. To investigate how recruitment is related to abundance and how climate affects recruitment at different times of the year we subdivided the population into three cohorts (juvenile, young, adult) based on snout vent length (SVL). Based on the incubation time and lizard growth rates we can estimate the approximate egg lay date and hatching date of the lizards caught during the census. Eggs take on average ~44 days to hatch, hatchlings are born SVL~15mm and grow at a rate of 0.17mm/day [1]. Juveniles were defined as lizards <35mm, approximately <60 days old at time of census and are an indication of late-wet season recruitment; eggs laid September, hatched October. Young lizards are individuals 35-44mm, <140 days old and indicative of mid-wet season recruitment (eggs laid July, hatched September). Adults were individuals >44mm, >140 days old and indicative of early-wet season recruitment (eggs laid May, hatched in July). Because very few eggs are laid during the dry season and annual survival is low (~2%), the majority of adults at the time of the census are predominantly early wet season recruits [2,3]. Log abundance was positively related to the log number of adults (y=0.4+1.03x, $F_{1,36}$ = 6.58, p = 0.014, r^2 = 0. 13), young (y=-1.18+0.86x, $F_{1,36}$ = 6.58, p = 0.014, r^2 = 0. 13) and juveniles (y=0.6+1.18x, $F_{1,36}$ = 6.58, p = 0.014, r^2 = 0. 13) (Figure S1).

Population growth rate

To investigate any density dependence effects on abundance we calculated population growth rate (PGR) using;PGR = $\log(N_{t+1}/N_t)$, [4]. Where N_t is abundance at time t and N_{t-1} is the previous year's abundance (Figure S2). There as no evidence of a partial- or auto- correlation in the population growth rate. Log abundance, log number of adults and log number of juveniles were positively related to population growth rate (abundance: $F_{1,38}$ = 5.18, p = 0.02; adults: $F_{1,36}$ = 6.58, p = 0.01; juveniles: $F_{1,36}$ = 5.22, p = 0.02) , but log number of young was not ($F_{1,36}$ = 1.81, p = 0.18) (Figure S1).

References

1. Andrews RM (1976) Growth-rate in island and mainland Anoline lizards. Copeia 1976: 477-482.
2. Andrews RM, Nichols JD (1990) Temporal and spatial variation in survival rates of the tropical lizard *Anolis limifrons*. Oikos 57: 215-221.
3. Andrews RM, Rand AS, Guerrero S (1982) Seasonal and spatial variation in the annual cycle of a tropical lizard. In: Rhodin AGJ, Miyata K, editors. Advances in Herpetology and Evolutionary Biology A fetschrift for Ernest E Williams. Cambridge, MA: Museum of Comparative Zoology.
4. Sibly RM, Hone J (2002) Population growth rate and its determinants: an overview. Philosophical Transactions of the Royal Society of London B 257: 1149-1170.
