# Supplementary material for: Long-Term Data Reveal a Population Decline of the Tropical Lizard Anolis apletophallus, and a Negative Affect of El Nino Years on Population Growth Rate
Source: PLoS One. 2015 Feb 11;10(2):e0115450. doi: 10.1371/journal.pone.0115450 (PMC4325001; doi:10.1371/journal.pone.0115450)
Supplement: S2 Supporting Information — Description of the climate indices and an analysis of how these have changed over time and how these are correlated. (PDF) [file pone.0115450.s021.pdf]

Supporting Information S2: Climate Data.

Climate variables were calculated from temperature and rainfall data from BCI provided by the Terrestrial-Environmental Sciences Program of the Smithsonian Tropical Research Institute. We calculated climate change indices used previously by Aguilar et al [1] . Rainfall indices were calculated using RClimdex (http://etccdi.pacificclimate.org/software.shtml), but because of missing data, RClimdex could not be used to calculate indices for maximum and minimum temperature, instead these were calculated separately using custom scripts in R. The Southern Oscillation Index (SOI, National Centre for Atmospheric Research, http://www.cgd.ucar.edu/cas/catalog/climind/soi.html) was also included as a climate variable. Linear regression of raw (untransformed) data was used to investigate if climate variables have changed with time (Table S2, S3) and how SOI was related climate variables (Table S2.2, Figures S2.4, S2.5)

References:

1. Aguilar E, Peterson TC, Obando PR, Frutos R, Retana JA, et al. (2005) Changes in precipitation and temperature extremes in Central America and northern South America, 1961-2003. Journal of Geophysical Research: Atmospheres 110: D23107.

Table S2.1. Climate change indices a) taken from [1] and b) indicies designed specifically for this study.

a)

ID	Indicator name	Definitions	UNITS
TXx	Max Tmax	Monthly maximum value of daily maximum temp	ºC
TNx	Max Tmin	Monthly maximum value of daily minimum temp	ºC
TXn	Min Tmax	Monthly minimum value of daily maximum temp	ºC
TNn	Min Tmin	Monthly minimum value of daily minimum temp	ºC
TN10p	Cool nights	Percentage of days when TN<10th percentile	Days
TX10p	Cool days	Percentage of days when TX<10th percentile	Days
TN90p	Warm nights	Percentage of days when TN>90th percentile	Days
TX90p	Warm days	Percentage of days when TX>90th percentile	Days
DTR	Diurnal temperature range	Monthly mean difference between TX and TN	ºC
RX1day	Max 1-day precipitation amount	Monthly maximum 1-day precipitation	Mm
Rx5day	Max 5-day precipitation amount	Monthly maximum consecutive 5-day precipitation	Mm
SDII	Simple daily intensity index	Annual total precipitation divided by the number of wet days (defined as PRCP>=1.0mm) in the year	Mm/day
R10	Number of heavy precipitation days	Annual count of days when PRCP>=10mm	Days
R20	Number of very heavy precipitation days	Annual count of days when PRCP>=20mm	Days
CDD	Consecutive dry days	Maximum number of consecutive days with RR<1mm	Days
CWD	Consecutive wet days	Maximum number of consecutive days with RR>=1mm	Days
R95p	Very wet days	Annual total PRCP when RR>95th percentile	Mm
R99p	Extremely wet days	Annual total PRCP when RR>99th percentile	mm
PRCPTOT	Annual total wet-day precipitation	Annual total PRCP in wet days (RR>=1mm)	mm

b)

ID	Indicator name	Definitions	UNITS
Tmax>PBT	Days exceeding preferred body temperature	Number of days maximum temperature exceeds *Anolis apletophallus* preferred bidyt temperature (27^{0}C)	Days
MWT	Maximum wet season temperature	Average TX for June, July and August	ºC
MDT	Maximum dry season temperature	Average TX for January, February and March	ºC

Table S2.2. Results table of linear relationships between climate change indices and time from 1971 – 2011 (untransformed data). Bold indicates significant ($p<0.05$) changes through time.

	Slope	SE	r^2	p-value
Total precipitation	9.60	7.20	0.01	0.19
**Rainfall intensity**[+]	**0.14**	**0.05**	**0.14**	**0.009**
Heavy precipitation days[+]	0.03	0.15	-0.02	0.83
Very heavy precipitation days[+]	0.14	0.11	0.01	0.20
Consecutive dry days	-0.15	0.12	0.01	0.24
Consecutive wet days	0.05	0.06	-0.006	0.39
**Very wet days**	**5.54**	**5.46**	**0.15**	**0.007**
**Extremely wet days**	**6.39**	**2.13**	**0.16**	**0.004**
Wet season length	4.31	2.99	0.02	0.15
Wet season rainfall	3.62	2.26	0.03	0.11
Southern oscillation index (SOI)	0.007	0.01	-0.01	0.64
**Diurnal temperature range**[+]	**-0.14**	**0.02**	**0.39**	**<0.001**
**Maximum temperature**[+]	**-0.05**	**0.01**	**0.20**	**0.003**
**Minimum temperature**	**0.08**	**0.01**	**0.37**	**<0.001**
Percentage of warm days[+]	-0.002	0.001	0.04	0.12
Percentage of cool days[-]	-0.0004	0.001	-0.02	0.73
Percentage of warm nights[+]	0.001	0.001	0.01	0.20
**Percentage of cool nights**[-]	**-0.01**	**0.002**	**0.50**	**<0.001**
Days when max temperature above PBT (Tmax>PBT)	-0.002	0.002	-0.01	0.46
Max dry season temperature	-0.02	0.01	0.07	0.06
**Max wet season temperature**	**-0.07**	**0.01**	**0.26**	**0.008**

[+] increased significantly in the region [1]

[-] decreased significantly in the region [1]

Table S2.3 Relationship between southern oscillation index (SOI) with other climate variables (transformed and standardised variables). Text in bold indicates a significant linear relationship ($p<0.05$).

	Slope	SE	r^2	p-value
Total precipitation	0.28	0.15	0.05	0.07
**Rainfall intensity**	**0.30**	**0.15**	**0.06**	**0.05**
Heavy precipitation days	0.07	0.15	-0.02	0.64
Very heavy precipitation days	0.24	0.15	0.03	0.12
Consecutive dry days	-0.12	0.16	-0.01	0.43
Consecutive wet days	0.21	0.15	0.02	0.17
**Very wet days**	**0.34**	**0.15**	**0.09**	**0.03**
Extremely wet days	0.26	0.15	0.04	0.09
Wet season length	0.24	0.15	0.03	0.12
Wet season rainfall	0.06	0.15	-0.02	0.66
Diurnal temperature range	0.15	0.17	-0.001	0.36
Max temperature	-0.01	0.17	-0.02	0.97
**Min temperature**	**-0.40**	**0.16**	**0.13**	**0.01**
Percentage of warm days	-0.01	0.17	-0.02	0.94
**Percentage of cool nights**	**0.46**	**0.15**	**0.18**	**0.005**
**Percentage of warm nights**	**-0.65**	**0.13**	**0.39**	**<0.001**
Percentage of cool days	0.19	0.17	0.008	0.26
Days when max temperature above PBT (Tmax>PBT)	0.25	0.16	0.03	0.13
Max dry season temperature	-0.08	0.17	-0.02	0.61
Max wet season temperature	0.003	0.17	-0.02	0.98
