# Supplementary material for: Long-Term Data Reveal a Population Decline of the Tropical Lizard Anolis apletophallus, and a Negative Affect of El Nino Years on Population Growth Rate
Source: PLoS One. 2015 Feb 11;10(2):e0115450. doi: 10.1371/journal.pone.0115450 (PMC4325001; doi:10.1371/journal.pone.0115450)
Supplement: S3 Supporting Information — Detailed description of the cross-correlation analysis and information theoretic modelling procedure (PDF) [file pone.0115450.s022.pdf]

Supporting Information S3. Data analysis

Temporal trends, covariation and cross-correlations

We plotted temporal trends of abundance and population growth rate with climate variables (Figure S3.1-3.4). To investigate possible cycles in abundance, population growth rate and climate, and any covariation in these variables we used autocorrelation (ACF), partial autocorrelation (PACF) and cross-correlation analyses. Autocorrelation is the cross correlation of a variable with itself at two points in time. Partial autocorrelation is the autocorrelation between two observations after removing any linear dependence between them due to other confounding variables. Cross correlation provides an estimate of the correlation between two times series at different time lags. If there were significant correlations, larger than the confidence intervals (dashed lines) with negative lag, *x* is said to leading *y*, by lag *h*.

Although large fluctuations in abundance were observed across the 40-years, there was little evidence of significant long-term cycles in abundance. Auto- and partial autocorrelation analysis identified 1-year lag in abundance, i.e. abundance in any year was positively correlated to abundance in the previous year (Figure S38) In contrast, a 7-year cycle in population growth rate was observed (Figure S8), population growth rate at time *t0* was correlated with population growth rate *t7* and *t15*.

Abundance in any year (N_i) was positively correlated with abundance in the previous year (N_{i-1}) (Linear regression: (N_{i-1}) (r^2= 0.27, $F_{1,38}$ = 16.11, $p < 0.001$, y = 16.6+0.53x). Such serial correlation is a common pattern in many taxa [1]. To account for this serial autocorrelation in the data we included an autoregressive term to all climate models ($AR_1 * N_{i-1}$). The AR term, estimated by fitting an autoregressive model was 0.57, and this term preceded the climate variables of interest ($x_1, x_2, \ldots x_n$) as so;

$N_i = 0.57 * N_{i-1} + x_{1..n}$.

Modelling Procedure

To investigate how climate relates to lizard abundance on BCI we used the information theoretic (IT) model selection approach based on Akiake's Information Criterion (AIC) as outlined in Burnham and Anderson (2002). The IT model selection approach is a useful way to identify the most likely model(s) given the data [2,3] and has several advantages over other step-wise procedures, which have been outlined in detail elsewhere [e.g. 3,4]. Most importantly, it provides an estimate of the weight of evidence in favour of a model being the best model, out of the set of candidate models considered [5]. This model weight can be simply interpreted as the probability that a given model, within a set, is the best approximating model. This approach does not simply compare one model against a null hypothesis (like traditional hypothesis testing), but compares amongst competing models. The Akiake weights also have the added advantage of providing evidence of model selection uncertainty, which is often ignored when attempting to choose a single best model. An akiake weight close to 1 is good evidence of a single best model, however if models are poor, then the best model weights will be low and several models can share similarly low probabilities; suggesting model selection uncertainty.

The information theoretic approach is not without pitfalls and a common problem is overfitting (i.e. considering too many models or explanatory variables) [6].

Considering too many models can lead to an AIC-best model that includes a variable only spuriously related to the data. This problem was confirmed in a recent study testing for the effect of weather on population abundance using 492 population abundance time series from the Global Population Dynamics Database (GPDD) [7]. Knape and de Valpine [7] suggest that for studies of specific populations *a priori* hypotheses about which factors are important can reduce the number of variables included in the analysis and minimise problems of overfitting. We adopted this approach when choosing a candidate set of models.

Creating a candidate set of models

We included all climate variables previously associated with changes in abundance, climate variables that have changed over the last 40 years, and the global climate variable Southern Oscillation Index (SOI) in the previous year. To test the hypothesis that climate change places thermoregulatory constraints on shade-adapted rainforest lizards [8], we also included the number of days that the maximum temperature exceeds *A. apletophallus'* preferred body temperature (PBT) of 27.8ºC [9], dry season maximum temperature, and wet season maximum temperature as explanatory variables. In total this approach yielded 14 variables, some of which were highly correlated. To avoid problems of mutli-colinearity, we removed one variable from each of the three highly correlated pairs of variables (r>0.80, Figure S7). We retained 11 climate variables for analysis (Table 1). A normally distributed dummy variable that was uncorrelated with the response was also included as an explanatory variable in the analysis to determine if the model selection procedure was likely to include spurious variables within our confidence set. We also included a model with no climatic variables in the candidate set (i.e. intercept only or Autoregressive term (AR) only) to compare the residual variance (σ^2) between models with and without the addition of a climate variable. Prior to analysis all the variables were checked for normality and transformed, where necessary, using the Box Cox Power transformations. All explanatory variables were centred and standardised by subtracting the mean and dividing by the standard deviation. To minimize problems associated with overfitting only one climatic variable was included in each model. For the response variable log abundance a total of 14 models were fitted (11 climate variables, 1 dummy variable, 1 AR model, 1 intercept model).

To model how climate affects each cohort we considered the weather conditions pertinent to that cohort, i.e. for the log number of juveniles we calculated climate variables for September-December, for log number of young, July-December, and for log number of adults, May-December. In some cases it was not appropriate to subset the climate variable because the variable was already a subset, e.g. wet season rainfall, wet/dry season maximum temperature or because only an annual estimate was available (SOI). As a result only 6 of the 11 variables were included in this analysis of the separate cohorts (see Table 1), resulting in a total of 8 models tested (6 climate variables, 1 dummy variable, 1 intercept model).

IT model selection procedure

For analysis AIC was corrected for small sample size (also called the second-order criterion AIC_c), this is necessary when the ratio of the number of observations (*n)* to the number of parameters (*K*) is small (< 40)[5]. The corrected AIC was calculated using the following equation:

$AIC_c = AIC + ((2*K(K+1))/n-K-1)$

For simplicity we use AIC to denote AIC_c for the remainder of the paper. Model AIC was calculated and compared between the candidate models, the model with the lowest AIC (AIC_{min}) was considered the best fitting model and the relative change in AIC (Δ_I) between models was calculated using:

$\Delta_I = AIC_i - AIC_{min}$

To compare between *R* set of models the Akaike Weights (ω_i) were calculated as follows:

$$w_i = \frac{\exp\left(-\frac{1}{2}\Delta_i\right)}{\sum_{r=1}^{R}\exp\left(-\frac{1}{2}\Delta_r\right)}$$

This entire model set can then be reduced to a confidence set using an evidence ratio cut off of 1/8, thus models with evidence ratios greater than 0.13 are included [5]. The evidence ratio of the model *j* is calculated using the following equation.

$ER = \exp(-1/2\ \Delta_I)$

The Akiake weights are then recalculated for the confidence set and as such they sum to one. The direction and magnitude of the explanatory variable’s effect on the response term is based on the parameter estimate and its 95% Confidence Interval (CI). All analyses were carried out using the statistical package R [10].

References:

1. Sibly RM, Barker D, Denham MC, Hone J, Pagel M (2005) On the regulation of populations of mammales, birds, fish and insects. Science 309: 607-610.
2. Hobbs NT, Hilborn R (2006) Alternatives to statistical hypothesis testing in ecology: A guide to self teaching. Ecological Applications 16: 5-19.
3. Johnson JB, Omland KS (2004) Model selection in ecology and evolution. Trends in Ecology and Evolution 19: 101-108.
4. Whittingham MJ, Stephens PA, Bradbury RB, Freckleton RP (2006) Why do we still use stepwise modelling in ecology and behaviour? Journal of Animal Ecology 75: 1182-1189.
5. Burnham KP, Anderson DR (2002) Model selection and inference. A practical information-theoretic approach. New York: Springer-Verlag.
6. Anderson DR, Burnham KP (2002) Pitfalls when using Infromation-Theoretic methods. Journal of Wildlife Management 66: 912-918.
7. Knape J, de Valpine P (2011) Effects of weather and climate on the dynamics of animal population time series. Proceedings of the Royal Society B: Biological Sciences 278: 985-992.
8. Huey RB, Deutsch CA, Tewksbury JJ, Vitt LJ, Hertz PE, et al. (2009) Why tropical forest lizards are vulnerable to climate warming. Proceedings of the Royal Society B: Biological Sciences 276: 1939-1948.
9. Ballinger RE, Marion KR, Sexton OJ (1970) Thermal ecology of lizard, *Anolis limifrons* with comparative notes on 3 additional Panamanian Anoles. Ecology 51: 246-254.
10. R Development Core Team (2006) R: A language and environment for statistical computing. Vienna, Austria.
