# Supplementary material for: Long-Term Data Reveal a Population Decline of the Tropical Lizard Anolis apletophallus, and a Negative Affect of El Nino Years on Population Growth Rate
Source: PLoS One. 2015 Feb 11;10(2):e0115450. doi: 10.1371/journal.pone.0115450 (PMC4325001; doi:10.1371/journal.pone.0115450)
Supplement: S1 Table — (PDF) [file pone.0115450.s023.pdf]

Table S1. Description of climate change indices used in the study.

Climate change indices a) taken from (Aguilar et al. 2005) and b) indicies designed specifically for this study.

a)

ID	Indicator name	Definitions	UNITS
TXx	Max Tmax	Monthly maximum value of daily maximum temp	ºC
TNx	Max Tmin	Monthly maximum value of daily minimum temp	ºC
TXn	Min Tmax	Monthly minimum value of daily maximum temp	ºC
TNn	Min Tmin	Monthly minimum value of daily minimum temp	ºC
TN10p	Cool nights	Percentage of days when TN<10th percentile	Days
TX10p	Cool days	Percentage of days when TX<10th percentile	Days
TN90p	Warm nights	Percentage of days when TN>90th percentile	Days
TX90p	Warm days	Percentage of days when TX>90th percentile	Days
DTR	Diurnal temperature range	Monthly mean difference between TX and TN	ºC
RX1day	Max 1-day precipitation amount	Monthly maximum 1-day precipitation	Mm
Rx5day	Max 5-day precipitation amount	Monthly maximum consecutive 5-day precipitation	Mm
SDII	Simple daily intensity index	Annual total precipitation divided by the number of wet days (defined as PRCP>=1.0mm) in the year	Mm/day
R10	Number of heavy precipitation days	Annual count of days when PRCP>=10mm	Days
R20	Number of very heavy precipitation days	Annual count of days when PRCP>=20mm	Days
CDD	Consecutive dry days	Maximum number of consecutive days with RR<1mm	Days
CWD	Consecutive wet days	Maximum number of consecutive days with RR>=1mm	Days
R95p	Very wet days	Annual total PRCP when RR>95th percentile	Mm
R99p	Extremely wet days	Annual total PRCP when RR>99th percentile	mm
PRCPTOT	Annual total wet-day precipitation	Annual total PRCP in wet days (RR>=1mm)	mm

b)

ID	Indicator name	Definitions	UNITS
Tmax>FBT	Days exceeding field body temperature	Number of days maximum temperature exceeds *Anolis apletophallus* field body temperature (27^{0}C)	Days
MWT	Maximum wet season temperature	Average TX for June, July and August	ºC
MDT	Maximum dry season temperature	Average TX for January, February and March	ºC
