# Supplementary material for: Long-Term Data Reveal a Population Decline of the Tropical Lizard Anolis apletophallus, and a Negative Affect of El Nino Years on Population Growth Rate
Source: PLoS One. 2015 Feb 11;10(2):e0115450. doi: 10.1371/journal.pone.0115450 (PMC4325001; doi:10.1371/journal.pone.0115450)
Supplement: S2 Table — (PDF) [file pone.0115450.s024.pdf]

Table S2. Results table of linear relationships between climate change indices and time from 1971-2011. Uuntransformed data, bold indicates significant ($p<0.05$) changes through time.

	Slope	SE	r^2	p-value
Total precipitation	9.60	7.20	0.01	0.19
**Rainfall intensity**$^+$	**0.14**	**0.05**	**0.14**	**0.009**
Heavy precipitation days$^+$	0.03	0.15	-0.02	0.83
Very heavy precipitation days$^+$	0.14	0.11	0.01	0.20
Consecutive dry days	-0.15	0.12	0.01	0.24
Consecutive wet days	0.05	0.06	-0.006	0.39
**Very wet days**	**5.54**	**5.46**	**0.15**	**0.007**
**Extremely wet days**	**6.39**	**2.13**	**0.16**	**0.004**
Wet season length	4.31	2.99	0.02	0.15
Wet season rainfall	3.62	2.26	0.03	0.11
Southern oscillation index (SOI)	0.007	0.01	-0.01	0.64
**Diurnal temperature range**$^+$	**-0.14**	**0.02**	**0.39**	**<0.001**
**Maximum temperature**$^+$	**-0.05**	**0.01**	**0.20**	**0.003**
**Minimum temperature**	**0.08**	**0.01**	**0.37**	**<0.001**
Percentage of warm days$^+$	-0.002	0.001	0.04	0.12
Percentage of cool days$^-$	-0.0004	0.001	-0.02	0.73
Percentage of warm nights$^+$	0.001	0.001	0.01	0.20
**Percentage of cool nights**$^-$	**-0.01**	**0.002**	**0.50**	**<0.001**
Days when max temperature above PBT (Tmax>PBT)	-0.002	0.002	-0.01	0.46
Max dry season temperature	-0.02	0.01	0.07	0.06
**Max wet season temperature**	**-0.07**	**0.01**	**0.26**	**0.008**

$^+$ increased significantly in the region (Aguilar et al. 2005)

$^-$ decreased significantly in the region (Aguilar et al. 2005)
