# Supplementary material for: Long-Term Data Reveal a Population Decline of the Tropical Lizard Anolis apletophallus, and a Negative Affect of El Nino Years on Population Growth Rate
Source: PLoS One. 2015 Feb 11;10(2):e0115450. doi: 10.1371/journal.pone.0115450 (PMC4325001; doi:10.1371/journal.pone.0115450)
Supplement: S3 Table — (PDF) [file pone.0115450.s025.pdf]

Table S3. Relationship between Southern Oscillation Index (SOI) and other climate variables from 1971-2011. Climate variables transformed and standardised variables, text in bold indicates a significant linear relationship ($p<0.05$).

	Slope	SE	r^2	p-value
Total precipitation	0.28	0.15	0.05	0.07
**Rainfall intensity**	**0.30**	**0.15**	**0.06**	**0.05**
Heavy precipitation days	0.07	0.15	-0.02	0.64
Very heavy precipitation days	0.24	0.15	0.03	0.12
Consecutive dry days	-0.12	0.16	-0.01	0.43
Consecutive wet days	0.21	0.15	0.02	0.17
**Very wet days**	**0.34**	**0.15**	**0.09**	**0.03**
Extremely wet days	0.26	0.15	0.04	0.09
Wet season length	0.24	0.15	0.03	0.12
Wet season rainfall	0.06	0.15	-0.02	0.66
Diurnal temperature range	0.15	0.17	-0.001	0.36
Max temperature	-0.01	0.17	-0.02	0.97
**Min temperature**	**-0.40**	**0.16**	**0.13**	**0.01**
Percentage of warm days	-0.01	0.17	-0.02	0.94
**Percentage of cool nights**	**0.46**	**0.15**	**0.18**	**0.005**
**Percentage of warm nights**	**-0.65**	**0.13**	**0.39**	**<0.001**
Percentage of cool days	0.19	0.17	0.008	0.26
Days when max temperature above PBT (Tmax>PBT)	0.25	0.16	0.03	0.13
Max dry season temperature	-0.08	0.17	-0.02	0.61
Max wet season temperature	0.003	0.17	-0.02	0.98
