# Supplementary material for: Long-Term Data Reveal a Population Decline of the Tropical Lizard Anolis apletophallus, and a Negative Affect of El Nino Years on Population Growth Rate
Source: PLoS One. 2015 Feb 11;10(2):e0115450. doi: 10.1371/journal.pone.0115450 (PMC4325001; doi:10.1371/journal.pone.0115450)
Supplement: S4 Table — (PDF) [file pone.0115450.s026.pdf]

Table S4. Table of mean abundance, number of young, number of juveniles and number of adults from 1971-2011.

year	mean.abundance	no.young	no.juvenile	no.adult
1971	79	29	33	36
1972	56	12	18	17
1973	39	15	9	11
1974	44	9	9	8
1975	25	5	6	9
1976	168	15	11	29
1977	95	7	17	25
1978	152	18	16	45
1979	62	11	12	22
1980	54	7	7	17
1981	51.5	13	5	9
1982	28	14	7	11
1983	2.498	15	5	11
1984	1	6	6	12
1985	4.48	16	9	33
1986	27.7685	38	15	39
1987	43.129	NA	NA	NA
1988	50.066	NA	NA	NA
1989	92.679	NA	NA	NA
1990	79.3005	54	36	29
1991	24.7955	10	6	6
1992	14.39	6	2	4
1993	1	1	0	0
1994	13.8945	8	7	19
1995	7.453	3	2	2
1996	20.336	8	7	12
1997	37.6785	8	8	10
1998	10.9215	2	2	5
1999	6.9575	2	0	5
2000	48.084	9	9	20
2001	56.012	17	14	29
2002	49.075	9	9	19
2003	26.282	2	5	15
2004	17.363	3	5	7
2005	25.7865	7	3	10
2006	40.40375	8	8	19
2007	5.52055	2	2	13
2008	15.62875	0	2	14
2009	9.5341	1	4	5
2010	1.55655	1	2	2
2011	1.55655	2	2	0
